# Correlation Between the Number of Lenticulostriate Arteries and Imaging of Cerebral Small Vessel Disease

**DOI:** 10.3389/fneur.2019.00882

**Published:** 2019-08-12

**Authors:** Yuan-Chang Chen, Xiao-Er Wei, Jing Lu, Rui-Hua Qiao, Xue-Feng Shen, Yue-Hua Li

**Affiliations:** Institute of Diagnostic and Interventional Radiology, Shanghai Jiao Tong University Affiliated Sixth People's Hospital, Shanghai, China

**Keywords:** intracranial arterial diseases, cerebral small vessel disease, lenticulostriate arteries, magnetic resonance imaging, computed tomography perfusion, digital subtraction angiography

## Abstract

**Background and purpose:** Hypoperfusion plays an important role in the pathophysiology of cerebral small vessel disease (SVD). Lenticulostriate arteries (LSAs) are some of the most important cerebral arterial small vessels. This study aimed to investigate whether the number of LSAs was associated with the cerebral perfusion in SVD patients and determine the correlation between the number of LSAs and SVD severity.

**Methods:** Five hundred and ninety-four consecutive patients who underwent digital subtraction angiography were enrolled in this study. The number of LSAs was determined. Computed tomography perfusion (CTP) was used to calculate the cerebral blood flow (CBF), cerebral blood volume (CBV), mean transit time (MTT), and time to peak (TTP). Magnetic resonance imaging (MRI) was performed to assess cerebral infarct, cerebral microbleeds (CMBs), white matter hyperintensities (WMHs), enlarged perivascular spaces (EPVSs), and lacunes. An SVD compound score was calculated to express the level of cerebral SVD load.

**Results:** The SVD scores were negatively correlated with the number of the LSAs (*P* < 0.001, *r*_s_ = −0.44). The number of LSAs was inversely associated with the presence of any type of SVD (*P* < 0.001). The adjusted ORs of the SVD severity were 0.31 for LSA group 1 (LSA > 20) vs. group 2 (LSA = 10–20) and 0.47 for LSA group 2 (LSA = 10–20) vs. group 3 (LSA < 10). MTT and TTP were significantly higher and CBF was significantly lower when the number of LSAs was between 5 and 10 on each side of the basal ganglia (*P* < 0.001, <0.001, and <0.001, respectively). The CBV was slightly lower when the number of LSAs was between 5 and 10, while it was significantly lower when the number was <5 on each side of the basal ganglia (*P* < 0.05, <0.0001, respectively).

**Conclusion:** LSA count was lower in SVD patients than the non-SVD participants and there was a positive correlation between the cerebral perfusion and the number of LSAs. The LSA number was negatively associated with SVD severity, hypoperfusion might play an important role. This finding may have potentially important clinical implications for monitoring LSA in SVD patients.

## Introduction

Cerebral small vessel disease (SVD) is an intrinsic disorder of the small vessels of the brain including the small arteries, arterioles, venules, and capillaries (1). There are six closely correlated features of SVD on brain magnetic resonance imaging (MRI), including recent small subcortical infarct, white matter hyperintensities (WMHs), lacunes, cerebral microbleeds (CMBs), enlarged perivascular spaces (EPVSs), and atrophy (2). However, neuroimaging only reveals brain parenchyma lesions and does not directly reflect the processes and severity of SVD.

Cerebral arterial small vessels have two origins: superficially, they stem from the subarachnoid circulation as the terminal vessels of medium-sized arteries; deeper, from the base of the brain, they stem directly from the large vessels as arterial perforators (1). These perforating vessels are essential for maintenance of optimum functioning of the brain's most metabolically active nuclei and complex white matter networks (2). Lenticulostriate arteries (LSAs) are some of the most important vascular structures in the human brain and the sites of many neurologic diseases (3). Ischemic and hemorrhagic cerebral strokes often occur in the areas of the brain supplied by these perforating arteries (4). Lacunar infarcts account for 20% of all strokes, and the basal ganglia are involved in 35–44% of intracerebral hemorrhages (5). Thus, *in vivo* imaging of LSAs could provide important insights and help us understand the pathophysiology and mechanism of SVD. Usually, digital subtraction angiography (DSA) is considered the gold standard when visualizing smaller arteries, including the perforating arteries (5, 6).

Hypoperfusion is suggested to play an important role in the pathophysiology of SVD (7, 8). In addition, hypoperfusion may be particularly detrimental to neurons in the presence of capillary dysfunction or arteriolar disease, owing to concomitant impaired vasoreactivity (9), blood-brain barrier dysfunction (10), and less efficient extraction of oxygen and other diffusible nutrients (11). However, these associations are still debatable (8, 12, 13).

In our previous study (14), we found that the numbers of LSA stems in patients with hypertension were significantly lower than those in non-hypertensive volunteers, determined by 3D-TOF-MRA. Hypertension is one of the most important risk factors for SVD (15). On the basis of these results, we hypothesized that the number of LSAs may be associated with SVD severity. The aim of the current study was to investigate whether the number of LSAs was associated with cerebral perfusion in SVD and to determine the relationship between the number of LSAs and SVD severity.

## Materials and Methods

### Study Population/Patients

Between June 2014 and May 2018, 632 consecutive patients suspected of having arteriostenosis, intracranial aneurysm, arteriovenous malformation, or subarachnoid hemorrhage with corresponding neurological symptoms visited our hospital to undergo DSA. The inclusion criteria were as follows: patients who underwent CT, CTP, MRI, and DSA within 2 weeks of symptom onset; those between 18 and 80 years of age; those with no critical medical conditions; and those with no history of head trauma or tumors. The exclusion criteria were as follows: patients who did not undergo CT, CTP, and MRI; patients with contraindications for CTP and MRI; those with poor quality imaging data; patients with hemorrhagic stroke (based on CT), acute severe infarction or infarction involving the basal ganglia region (based on DWI), and intracranial aneurysm located in the middle cerebral artery that could have affected the origin of LSA. Any patients with other possible sources of white matter hypoattenuation on a chart review—such as multiple sclerosis, acute disseminated encephalomyelitis (ADEM), vasculitis, or connective tissue diseases—were also excluded. Ultimately, 594 patients were enrolled for further analysis.

This study was carried out in accordance with the recommendations of institutional guidelines. The protocol was approved by the committee of Shanghai Jiao Tong University Affiliated Sixth People's Hospital institutional review board. All subjects provided written informed consent in accordance with the Declaration of Helsinki. This study adhered to standard biosecurity and institutional safety procedures.

### DSA Examination

An interventional neuroradiologist performed DSA. Conventional 2D-DSA was performed on a monoplanar digital angiography unit (Axiom Artis VB22N; Siemens, Erlangen, Germany) with a 1,024 × 1,024 matrix and a 17–20 cm FOV. The contrast medium was injected at a flow rate of 4–5 mL/s and 2–3 mL/s in two projections; therefore, a total of 10 mL medium was injected into the internal carotid artery, and 7 mL was injected into the vertebral artery. Imaging data were transferred to a workstation (syngoXWP VA70B, Siemens). Then, we counted the number of visible LSA stems. Patients were divided into three groups (1, 2, and 3) according to the number of LSAs on both sides (>20, 10–20, <10) and on one side (>10, 5–10, <5).

### MRI Examination and Analysis

MRI scans were performed using a 3.0T MRI system (MAGNETOM Skyra 3.0T, Siemens, Amberg, Germany). MRI images were obtained parallel to the orbitomeatal line, using the following parameters: (i) TR/TE, 5120/62 ms; slice thickness, 4 mm; FOV, 220 × 220 mm; and three different directions of diffusion gradient and two b values (0 and 1,000 mm^2^/s) for DWI. (ii) TR/TE, 7,500/81 ms; slice thickness, 4 mm; and FOV, and 220 × 220 mm for FLAIR imaging. (iii) TR/TE, 4,730/72 ms; slice thickness, 4 mm; and FOV, 220 × 220 mm for T2W. (iv) TR/TE, 28/20 ms; slice thickness, 1 mm; and FOV, 220 × 220 mm for susceptibility weighted imaging (SWI). No contrast material was administered.

A recent infarct was defined as a hyperintense area on DWI, with a corresponding reduced signal on the apparent diffusion coefficient image, with or without increased signal on T2-weighted imaging or FLAIR, which corresponded with a typical vascular territory. Recent small subcortical infarcts were defined as ovoid or rounded lesions with similar signal characteristics to recent infarcts but were small (>3 mm and <20 mm) in diameter, in the centrum semiovale, internal capsule, basal ganglia, or brainstem, and were carefully distinguished from WMHs (2). Cortical infarcts were defined as infarcts involving cortical adjacent subcortical tissue, or large (>20 mm) subcortical/striatocapsular lesions (16). Deep and periventricular WMHs were both coded according to the Fazekas scale from 0 to 3, using T2-weighted imaging and FLAIR (17). Lacunes were defined as ovoid or rounded lesions, small (>3 mm and <20 mm) in diameter, in the centrum semiovale, internal capsule, basal ganglia, or brainstem, of cerebrospinal fluid (CSF) signal intensity on T2-weighted imaging and FLAIR, generally with a hyperintense rim on FLAIR and no increased signal on DWI (16). CMBs were defined as small (<5 mm), homogeneous, round foci of low signal intensity on gradient echo images in the cerebellum, brainstem, basal ganglia, white matter, or cortico-subcortical junction, differentiated from vessel flow voids and mineral depositions in the globi pallidi (16). EPVSs were defined as small (<3 mm) punctate (if perpendicular) and linear (if longitudinal to the plane of scan) hyperintensities on T2-weighted imaging in the centrum semiovale or basal ganglia, and they were rated on a validated semiquantitative scale from 0 to 4 (18). In this study, we only counted CMBs and EPVSs in the basal ganglia because in this region, they seem specifically associated with SVD (19, 20).

### Determining the Total MRI Burden of SVD

An SVD compound score, expressing the level of cerebral SVD load, was calculated according to the method described below. Based on the recently described score (18), we rated the total MRI burden of SVD on an ordinal scale from 0 to 4 by counting the presence of the following: lacunes and CMBs, which were defined as the presence of one or more lacunes (1 point if present) or any CMB (1 point if present); EPVSs were counted if they were moderate-to-severe (grade 2–4) in the basal ganglia (1 point if present); presence of WMHs was defined as either (early), confluent, deep WMH (Fazekas score 2 or 3) or irregular, periventricular WMH extending into the deep white matter (Fazekas score 3) (1 point if present) (21). The existence of CMBs, high-grade WMHs (HWHs), high-grade EPVSs (HPVSs), and lacunes were determined outside the acute infarct area (based on DWI), and two neurologists who were blinded to patients' clinical information independently investigated these lesions. The interobserver agreement values for the presence of CMBs, HWHs, HPVSs, and lacunes were 0.912, 0.956, 0.938, and 0.888, respectively (all *p* < 0.05). Any disagreement regarding the presence of SVD was resolved by consensus.

### CT and CTP Examination

The CT stroke protocol was performed on a Brilliance 256-channel iCT device (Brilliance iCT, Philips Medical Systems, Haifa, Israel). Components included pre- and post-contrast head CT from the skull base to the vertex with the following imaging parameters: 120 kilovolt (peak), 340 mA, 4 × 5 mm or 8 × 5 mm collimation, 1 s/rotation, and table speed of 15 mm/rotation. CTP comprised 2 phases with the following parameters: 80 kVp, 190 mA, 3–5 s delay, injection of 0.5 mL/kg (30–50 mL) iohexol (300 mg I/mL, Omnipaque; Nycomed, Princeton, NJ) at 4 mL/s. The initial phase consisted of a 45 s cine scanning at 1 rotation/s. A second phase was added with 1 rotation/s every 15 s for an additional 75 s.

All CTP studies covered a 20–40-mm slab with 4–8 sections measuring 5 mm each, centered between the anterior commissure and the centrum semiovale. Arterial and venous time-enhancement curves were obtained from the anterior cerebral artery and the superior sagittal sinus, respectively. CTP software (Intellispace portal, Philips Medical Systems, Haifa, Israel) was used to calculate the CBF, CBV, MTT, and TTP by deconvolution of 2 × 2 pixels. Arterial input and venous output functions were obtained from the anterior cerebral artery and from the superior sagittal sinus, respectively. Partial volume averaging of the arterial input curve was corrected by using the venous time-enhancement curve. Perfusion-weighted (PW) maps were calculated by averaging cine images over the duration of the first contrast passage through the brain.

**Figure 1:**
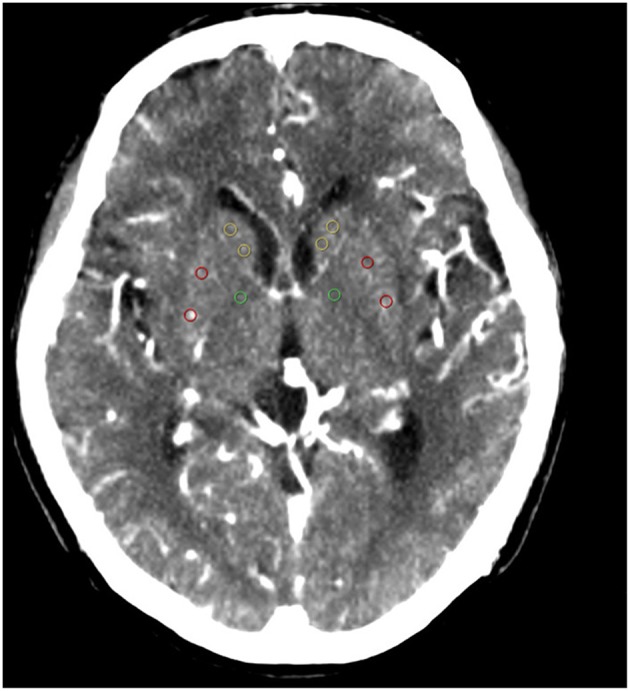
ROIs: (1) yellow ROIs are in the caudate heads; (2) red ROIs are in the putamen; (3) green ROIs are in the genu of the internal capsule. ROI, region of interest.

Functional maps (CBF, CBV MTT, and TTP) were imported into custom software (Intellispace portal, Philips Medical Systems, Haifa, Israel) for analysis. The region of interest (ROI) and automatic threshold-based techniques were used to collect data from the functional maps. Analysis was performed at the level of the superior portion of the basal ganglia regions for all patients according to the template shown in Figure 1. This region is routinely covered irrespective of the obtained CTP coverage (2–4 cm). Equal sized circular ROI (36 pixels) were placed in the basal ganglia regions on the PW map in every patient. Each ROI was enumerated to provide information on specific locations. Five ROIs were placed as follows: two in the head of the caudate, two in the putamen, and one in the genu of the internal capsule (Figure 1). ROI placements were independently verified by an experienced neuroradiologist (blinded to the patient details) to ensure that no CSF or gray matter structures were included. To minimize partial volume effects, we placed the ROIs at least 2 mm from tissue boundaries. CBF and CBV thresholds of >100 mL^−1^·min^−1^·100 g^−1^ or >8 mL·100 g^−1^, respectively, were applied to perfusion maps to minimize vascular-pixel contribution. A semiautomated threshold technique based on Hounsfield units was used to create a whole gray matter mask on the PW images. This mask was then applied to the functional maps to derive gray matter mean CBF, CBV, MTT, and TTP (22). The values for the 2 ROIs in the head of the caudate and the 2 ROIs in the putamen were averaged before they were entered into the analysis.

### Clinical and Laboratory Variables

Patients' data for traditional vascular risk factors (23) and previous episodes of stroke were collected. Hypertension was diagnosed as present when the patient had a resting systolic/diastolic blood pressure of ≥140/90 mm Hg in repeated measurements or had been taking oral antihypertensive agents. Diabetes mellitus was defined as a fasting blood glucose level of ≥7.0 mmol/L or treatment with oral hypoglycemic medications or insulin. Hyperlipidemia was defined as a total cholesterol level of ≥6.2 mmol/L, a low-density lipoprotein cholesterol level of ≥4.1 mmol/L, or if the patient had been treated with lipid-lowering medication after a diagnosis of hyperlipidemia at admission. Current smokers or those who had stopped smoking within 1 year before the index stroke were considered smokers. Previous stroke was defined if the patient had a previous stroke-like symptom combined with an ischemic lesion confirmed by brain imaging, and a history of TIA was excluded. We further collected data relating to the patients' prior medication; coronary artery disease including history of myocardial infarction, unstable angina, or angiographically confirmed occlusive coronary artery disease, and metabolic syndrome.

### Statistical Analyses

Continuous data were analyzed using the analysis of variance (ANOVA) test. Univariate analysis was performed using the χ^2^ test for categorical data. A trend χ^2^ test and Spearman rank correlation analysis were used to examine evidence of association between the number of LSAs and severity of the MRI makers of SVD. A Mann–Whitney U-test was used to compare the number of LSAs and SVD scores among the different severity groups based on each SVD MRI markers. Multivariate analysis was performed using logistic regression analysis to investigate the association of the number of LSAs with the SVD severity. Multivariate analysis was adjusted for age, sex, hypertension, diabetes mellitus, hyperlipidemia, previous stroke, smoking, coronary artery disease, and metabolic syndrome. The results were expressed as adjusted ORs (multivariate analysis) together with their 95% confidence intervals (CIs). ANOVA was performed to compare the cerebral perfusion in the basal ganglia region with different number of LSAs. A *p* < 0.05 was considered significant. The statistical packages SPSS 17.0 (IBM Corp., Armonk, NY, USA) was used for the analysis.

## Results

**Figure 2:**
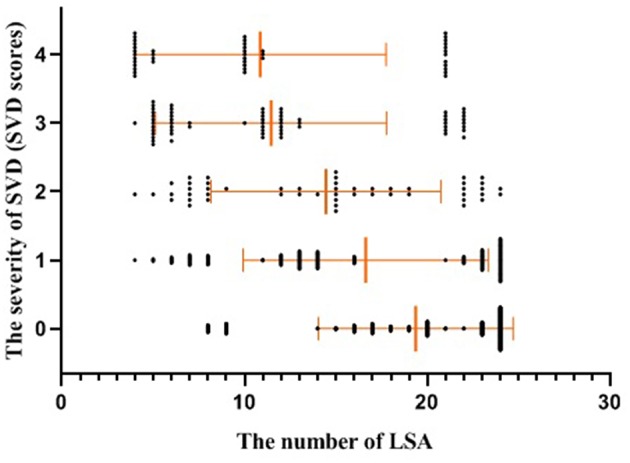
Scatter plot with mean and standard deviation showed the number of the LSAs was inversely associated with the severity of SVD (SVD scores). SVD, small vessel disease; LSAs, lenticulostriate arteries.

The baseline characteristics of the study population (*n* = 594) are presented in Table 1. The mean age of patients was 64.21 ± 10.26 years and 61.62% were male. The prevalence of hypertension, previous stroke, smoking, and age were significantly different among the three groups according to LSA number on both sides (*P* < 0.001, <0.001, =0.04, <0.001, respectively) (Table 1). CMBs were found in 28.28% of the subjects, HWHs in 19.02%, HPVSs in 20.20%, and lacunes in 41.59%. Univariate analysis showed that reduction in the number of LSAs on both sides was negatively associated with the presence of each SVD marker (*P* < 0.001). The correlation coefficients (rs) were −0.67, −0.51, −0.42, and −0.61 for CMBs, HWHs, HPVSs, and lacunes, respectively. The number of the LSAs on both sides was negatively correlated with SVD scores (*P* < 0.001, rs = −0.44) (Table 2 and Figure 2). The adjusted ORs of the SVD severity were 0.31 (95% CI 0.19–0.50, *p* < 0.001) for LSA group 1 (LSA > 20) vs. group 2 (LSA = 10–20) and 0.47 (95% CI 0.30–0.72, *p* < 0.001) for LSA group 2 (LSA = 10–20) vs. group 3 (LSA < 10). MTT and TTP were significantly higher and CBF significantly lower in the LSA group 2 (LSA = 5–10) in each side of the basal ganglia. CBV was slightly lower in LSA group 2 (LSA = 5–10), while it was significantly lower in LSA group 3 (LSA < 5) in each side of the basal ganglia (Table 3).

**Table 1:** Baseline characteristics by the number of LSAs.

**Number of LSAs (both sides)**	**>20 (*n* = 247) *n* (%)**	**10–20 (*n* = 215) *n* (%)**	**<10 (*n* = 132) *n* (%)**	***P*-value**
**Demographic data**				
Male	154 (42.08)^*^	133 (36.34)	79 (21.58)	0.89
Female	93 (40.79)	82 (35.96)	53 (23.25)	
Age, years#	58.99 ± 7.30	65.60 ± 10.69	71.70 ± 8.86	<0.001
**Risk factors**				
Hypertension#	160 (36.45)	164 (37.36)	115 (26.20)	<0.001
Diabetes mellitus	72 (40.22)	66 (36.87)	41 (22.91)	0.91
Hyperlipidemia	25 (34.72)	28 (38.89)	19 (26.39)	0.42
Previous stroke#	31 (25.62)	47 (38.84)	43 (35.54)	<0.001
Smoking#	36 (32.43)	42 (37.84)	33 (29.73)	0.04
Coronary artery disease	39 (34.51)	41 (36.28)	33 (29.20)	0.09
Metabolic syndrome	113 (39.79)	106 (37.32)	65 (22.89)	0.70

LSAs, lenticulostriate arteries. Each baseline characteristic of the study population was compared among the three different LSA number groups on both sides. A P < 0.05 was considered significant (#).

* The data indicates the number for male participants in the group (LSA > 20) and the data in the bracket indicates the percentage of participants falling into that particular group (LSA > 20/total male participants). Similar for the other data.

**Table 2:** Comparison of the number of LSAs according to the presence of SVD and the severity of SVD.

**The number of LSA**			**>20**	**10–20**	**<10**	***P*-value**	**Mean value ± SD**	***P*-value**	**r_**s**_**
CMBs	–	(*n =* 426)	235 (55.16)^*^	163 (38.26)	28 (6.57)	<0.001	19.79 ± 4.78	<0.001	−0.67
	+	(*n =* 168)	12 (7.14)	52 (30.95)	104 (61.90)		9.26 ± 4.69		
HWHs	–	(*n =* 481)	232 (48.23)	180 (37.42)	69 (14.35)	<0.001	18.57 ± 5.71	<0.001	−0.51
	+	(*n =* 113)	15 (13.27)	35 (30.97)	63 (55.75)		9.34 ± 5.40		
HPVSs	–	(*n =* 474)	220 (46.41)	174 (36.71)	80 (16.88)	<0.001	18.25 ± 6.01	<0.001	−0.42
	+	(*n =* 120)	27 (22.50)	41 (34.17)	52 (43.33)		11.15 ± 6.36		
Lacunes	–	(*n =* 347)	194 (55.91)	136 (39.19)	17 (4.90)	<0.001	20.31 ± 4.43	<0.001	−0.61
	+	(*n =* 247)	53 (21.46)	79 (31.98)	115 (46.56)		11.90 ± 6.29		
**SVD SCORES**									
0		(*n =* 290)	141 (48.62)^#^	106 (36.55)	43 (14.82)		19.37 ± 5.33		
1		(*n =* 147)	66 (44.90)	52 (35.37)	29 (19.73)		16.63 ± 6.71		
2		(*n =* 51)	13 (25.49)	21 (41.18)	17 (33.33)		14.45 ± 6.29		
3		(*n =* 63)	15 (23.81)	22 (34.92)	26 (41.27)		11.46 ± 6.32		
4		(*n =* 43)	12 (27.91)	14 (32.56)	17 (39.53)	<0.001	10.86 ± 6.89	<0.001	−0.44

SVD, small vessel disease; LSAs, lenticulostriate arteries; CMB, cerebral microbleed; HWHs, high-grade white matter hyperintensities; HPVSs, high-grade enlarged perivascular spaces; SD, standard deviation; r_s_, correlation coefficient.

The number of LSAs was compared according to the presence of each SVD markers, including CMBs, HWHs, HPVSs, and lacunars, and according to the SVD scores (SVD severity). The LSA numbers were analyzed as both categorical variable and continuous variable on both sides.

* The data indicates the number of participants without CMBs in the group (LSA > 20) and the data in the bracket indicates the percentage of participants falling into that particular group (LSA > 20/total participants without CMBs). Similar for the other data.

# *The data indicates the number of participants with SVD score = 0 in the group (LSA > 20) and the data in the bracket indicates the percentage of participants falling into that particular group (LSA > 20/total participants with SVD score = 0). Similar for the other data*.

**Table 3:** Comparison of cerebral perfusion in the basal ganglia regions.

	**Left**			**Right**		
The number of LSAs	>10	5–10	<5	>10	5–10	<5
(each side)	*n =* 240	*n =* 212	*n =* 142	*n =* 251	*n =* 220	*n =* 123
**Internal capsule**						
MMT (s)	3.15 ± 0.23	4.23 ± 0.42^*^	4.96 ± 0.22^*^	4.96 ± 0.22	4.29 ± 0.38^*^	4.98 ± 0.23^*^
TTP (s)	9.15 ± 0.67	9.84 ± 0.65^*^	10.86 ± 0.69^*^	9.18 ± 0.61	9.91 ± 0.55^*^	10.95 ± 0.77^*^
CBF (ml 100 g^−1^.min^−1^)	47.88 ± 5.01	43.25 ± 5.36^*^	39.44 ± 4.88^*^	47.52 ± 4.74	42.68 ± 5.72^*^	39.64 ± 2.88^*^
CBV (ml 100 g^−1^)	2.49 ± 0.21	2.45 ± 0.18#	2.35 ± 0.28^*^	2.50 ± 0.23	2.46 ± 0.17#	2.33 ± 0.31^*^
**Head of the caudate**						
MMT (s)	3.08 ± 0.13	4.02 ± 0.22^*^	4.81 ± 0.34^*^	3.08 ± 0.18	4.06 ± 0.32^*^	4.88 ± 0.32^*^
TTP (s)	8.62 ± 0.69	9.60 ± 0.57^*^	10.48 ± 0.71^*^	8.67 ± 0.71	9.69 ± 0.53^*^	10.51 ± 0.57^*^
CBF (ml 100 g^−1^·min^−1^)	69.52 ± 5.48	63.48 ± 7.03^*^	59.17 ± 6.39^*^	68.98 ± 6.85	63.42 ± 7.03^*^	58.27 ± 4.91^*^
CBV (ml 100 g^−1^)	3.59 ± 0.46	3.52 ± 0.38#	3.34 ± 0.31^*^	3.58 ± 0.48	3.51 ± 0.35#	3.38 ± 0.33^*^
**Putamen**						
MMT (s)	3.09 ± 0.12	4.00 ± 0.26^*^	4.82 ± 0.35^*^	3.11 ± 0.18	4.03 ± 0.32^*^	4.89 ± 0.28^*^
TTP (s)	8.51 ± 0.67	9.50 ± 0.55^*^	10.34 ± 0.64^*^	8.61 ± 0.61	9.54 ± 0.52^*^	10.47 ± 0.68^*^
CBF (ml 100 g^−1^·min^−1^)	76.22 ± 8.26	68.49 ± 8.55^*^	64.22 ± 7.89^*^	76.50 ± 8.59	67.40 ± 9.15^*^	64.01 ± 6.67^*^
CBV (ml 100 g^−1^)	4.07 ± 0.24	4.01 ± 0.30#	3.87 ± 0.35^*^	4.03 ± 0.25	3.98 ± 0.29#	3.88 ± 0.31^*^

LSAs, lenticulostriate arteries; MTT, mean transit time; TTP, time to peak; CBF, cerebral blood flow; CBV, cerebral blood volume.

The cerebral perfusion indices were compared among the three LSA groups in three regions of the basal ganglia for each side.

* *P < 0.001*,

# *P < 0.05*.

## Discussion

In this study, we observed the LSAs by using DSA *in vivo* and found that the number of LSAs was lower in SVD patients than the non-SVD participants. The LSA number had a positive correlation with cerebral perfusion and a negative correlation with SVD severity.

Moody et al. found that brain microvascular density was reduced in subjects with leukoaraiosis (24). Our results indicated that the number of LSAs was low in SVD, which was consistent with their findings. In our patient dataset, presence of hypertension, advanced age, previous strokes, smoking, and coronary artery disease were higher in the group with fewer LSAs than the group with more LSAs. These results were similar to those reported in previous studies (15). In our previous study (14), we examined 60 patients with hypertension and 60 non-hypertensive volunteers with three dimension-time of flight -magnetic resonance angiography (3D-TOF-MRA) and found that the number of LSA stems was significantly low in hypertensive patients. These findings were likely because of microvascular rarefaction due to hypertension (25). Hypertension alters the structure and function of microcirculation. These changes in the microvascular network lead to a reduction in the number of arterioles or capillaries within the vascular beds of various tissues, known as vascular rarefaction (26).

Additionally, our findings showed that there were significantly fewer visible LSAs in the presence of more severe SVD. We thought that presumably the endothelial-function impairment of small vessels was minor in the early phase of SVD and narrowing of small arterioles may lead to a reduction in CBF (27), while cerebrovascular autoregulation maintains relatively constant CBF over a range of cerebral perfusion pressures (28). However, with SVD progression, diffuse cerebrovascular endothelial failure occurred, vessel walls thickened, and luminal narrowing became more severe, eventually leading to loss of normal autoregulatory ability in these vessels, which could contribute further to tissue damage (29) and concomitant CBF and CBV reduction (30). In the present study, we found that CBF was significantly lower and CBV was slightly lower when the number of LSAs was between 5 and 10; however, CBF and CBV were significantly lower when the number of LSAs was <5. A systematic review showed that CBF was negatively related to SVD severity (8). In ischemic lesions caused by SVD, WMH is considered a form of incomplete infarct owing to a state of chronic hypoperfusion of the white matter caused by vessel lumen restriction. Alternatively, occlusion of a small vessel is hypothesized to occur, leading to lacunar infarcts (1). Accordingly, our results may suggest that the LSA number was inversely correlated with SVD severity, and hypoperfusion might play an important role.

In this study, we chose LSA as a marker of SVD, because it has a relatively stable number of branches arising from the middle cerebral artery. The LSA is a terminal artery without collateral branches (31); it arises vertically from the middle cerebral artery, which renders the territory supplied by the LSAs more susceptible to ischemia and leads to high risk of arteriolar necrosis (32). Moreover, the LSAs primarily supply important territories including the basal ganglia and internal capsule, which are vulnerable to ischemia and cause obvious clinical symptoms (31).

The lenticulostriate territory supplies the upper part of the head and the body of the caudate nucleus, putamen, lateral part of the pallidum, superior parts of both the anterior and posterior limbs and genu of the internal capsule, and lateral third of the anterior commissure. We selected the head of the caudate nucleus, putamen, and genu of the internal capsule as ROIs, because these territories were in balance with the territories of other perforating arteries from the internal carotid, anterior cerebral, and anterior choroidal arteries, but not in balance with the territories of the large vessels (32). Perfusion in these regions would not be affected by blood flow in the large vessels.

There are 4 to 12 LSAs that arise from the proximal middle cerebral artery in its horizontal segment, and LSAs vary from 200 to 400 μm in size (33). The drop in LSA number could reflect rarefaction in the SVD process (26), which might be due to the decrease of the absolute number of LSAs or dip in arterial diameter to below the detection limit. DSA is the gold standard for diagnosis of cerebral vascular disease and the resolution of DSA is 90 μm (34). Hence, we considered that DSA could display most of the LSAs and the low arterial number was mainly due to the lower absolute arterial number. We could not rule out the differences in patient vascular anatomy, but we considered that the distribution of such differences might be random in our participants.

Our study has some limitations. First, a total SVD score was used in this study to evaluate the severity of SVD. Some may argue that there are differences in the underlying pathogenetic mechanisms leading to these different SVD features. However, all of these MRI features are considered to result from disease in the small vessels, and they often co-occur (21). The total SVD score provides a more complete overall view of the impact of SVD on the brain than do the individual MRI features separately. Second, we did not evaluate cerebral atrophy. Because a prior study reported a non-specific association between atrophy and SVD (35), it could have occurred in many other conditions including normal aging. Third, our controls did not represent “healthy” control comparisons, because DSA cannot be performed in completely healthy populations. Fourth, our patient selection favored patients who were comparatively less-disabled and able to undergo MRI. However, both these conditions could have probably led to an underestimation of the association between the total burden of SVD and the number of LSAs. Last, our study had a retrospective design, which is a substantial limitation for this study type; additionally, it is difficult to ascertain the causation between these factors in this cross-sectional study, so a longitudinal study will be performed in the future to determine if LSA number can be used as a marker for SVD severity.

In conclusion, the number of LSAs was lower in SVD patients and there was a positive correlation between cerebral perfusion and the number of LSAs in SVD. This study has shed light on the association of SVD severity and the number of LSAs, and hypoperfusion might play an important role. This finding may have potential important clinical implications for monitoring LSA in SVD patients.

## Data Availability

The datasets generated for this study are available on request to the corresponding author.

## Ethics Statement

This study was carried out in accordance with the recommendations of institutional guidelines given by the committee of Shanghai Jiao Tong University Affiliated Sixth People's Hospital institutional review board, and all subjects provided written informed consent. All subjects gave written informed consent in accordance with the Declaration of Helsinki. The protocol was approved by the committee of Shanghai Jiao Tong University Affiliated Sixth People's Hospital institutional review board.

## Author Contributions

Y-HL and Y-CC contributed to the conception and design of the study. Y-CC, R-HQ, and X-FS organized the database. X-EW and JL performed the statistical analysis. Y-CC wrote the first draft of the manuscript. Y-HL, Y-CC, X-EW, and JL wrote sections of the manuscript. All authors contributed to manuscript revision and read and approved the submitted version.

### Conflict of Interest Statement

The authors declare that the research was conducted in the absence of any commercial or financial relationships that could be construed as a potential conflict of interest.
